# Emotional impact of AI-generated vs. human-composed music in audiovisual media: A biometric and self-report study

**DOI:** 10.1371/journal.pone.0326498

**Published:** 2025-06-25

**Authors:** Nikolaj Fišer, Miguel Ángel Martín-Pascual, Celia Andreu-Sánchez

**Affiliations:** 1 Neuro-Com Research Group, Institut de Neurociències and Audiovisual Communication and Advertising Department, Universitat Autònoma de Barcelona, Cerdanyola del Vallès, Barcelona, Spain; 2 PhD Program in Sociology of Culture, University of Ljubljana, Ljubljana, Slovenia; 3 PhD Program in Audiovisual Communication and Advertising, Universitat Autònoma de Barcelona, Cerdanyola del Vallès, Barcelona, Spain; 4 Research and Development, Spanish Public Television Institute (RTVE), Sant Cugat del Vallès, Barcelona, Spain; Universidad Nacional de Tres de Febrero, ARGENTINA

## Abstract

Generative artificial intelligence (AI) has evolved rapidly, sparking debates about its impact on the visual and sonic arts. Despite its growing integration into creative industries, public opinion remains sceptical, viewing creativity as uniquely human. In music production, AI tools are advancing, yet emotional expression remains largely overlooked in development and research. This study examined whether AI-powered music creation can evoke the same emotional impact as human-created music in audiovisual contexts. Participants (*N* = 88) watched videos accompanied by different audio tracks across three conditions: human-created music (HCM), AI-generated music using more sophisticated and detailed keyword prompts (AI-KP) and AI-generated music using simpler and less detailed prompts based on discrete and dimensional emotional values (AI-DP). Biometric data and personal affective responses were registered during this process. The results show that both AI soundtracks led to wider pupil dilation compared with human-created music but did not differ significantly from each other. AI-generated music with sophisticated prompts (AI-KP) resulted in a higher blink rate and skin impedance level as markers of attention and cognitive load, while emotional valence remained consistent across conditions. Participants found AI-generated music more arousing that HCM, while HCM was perceived as more familiar than both AI conditions.

## Introduction

Can AI-generated music evoke the same emotional responses as human-composed music in audiovisual content? The development of generative artificial intelligence is a process that has been unfolding throughout the past few decades, yet the rapid progression seen in the recent years has resulted in the stimulation of numerous discussions regarding its effect on both visual [1,2] and sonic [2,3] artists. Regardless of its unavoidable integration within different spheres of creative media production, the opinions of wider audiences on AI-generated art, labelled as such, are not favourable, both in terms of musical pieces [4] and paintings [2] as well as in other domains of the creative industry [5]. Millet et al. [2] interpret this negative bias as creative value being an inherently and exclusively human trait. Similarly, users tend to demonstrate a much more open, extroverted, self-disclosing and agreeable behaviour during initial social interactions with human compared with AI chatbots [6].

However, many, if not most, industries have been integrating generative AI into their production process, including the field of audiovisual production. While scholars regard the Illiac Suite [7] as the first musical tool of this kind, contemporary generators have moved beyond score generation to operate within various aspects of music production, such as sound design, mixing, mastering etc. However, one of the studies of the current state of musical generative AI, analysing 118 available AI generators, showed that the role of emotion has been widely neglected, both in AI instruments and in academic reporting, with emotion being considered in only 18 out of the 118 generators analysed [8].

### AI and music

In recent years, there have been several studies that focussed on the impact that generative AI tools have on musical creators [9–15] and on consumers [9,11,12,15–17]. There is conflicting evidence regarding the ability of AI-generated music to reproduce emotional effects in the same way as human-made music does, with some authors [9,12] stating that it can effectively reproduce the emotional effects but others [11,16,17] assessing that it cannot. However, the output and emotional effect on the listener relies heavily on the language model used, how it is used and the underlying dataset [9,10,14,15]. Another important aspect of generative AI research is the method of evaluating the output. Since there is no standardised method for evaluating AI, scholars have proposed a unified evaluative approach to AI-generated music [10,12,14,18], consisting of both objective and subjective methods of evaluation, or a combined approach [18]. The problem identified regarding the use of AI in music, apart from the reproducibility of emotions and lack of emotional depth, originality and the impact on future jobs [10,11,15,17], is the effect that AI music curation and recommendation systems will have on listeners’ preferences and affective capabilities and how they will contribute to bias [16]. With language models being optimised and music generators being updated rapidly, one may anticipate that the affective properties of AI-generated music will become increasingly more similar to those of human-made music, as the detectability of who is the creator behind a musical track diminishes [5]. The studied literature also indicates a progressively narrowing gap between the creator and the consumer or user through the development of AI music generators.

### AI tools for generating audio

As of December 2024, there are many AI tools for generating audio, and some of the most relevant use text-to-music tools. Suno (Suno Inc.) is primarily a text-to-music generator based on a transformer model. However, with the fourth version being released mid-November 2024, its functionalities expanded to the generation of music based on one’s own audio input, remastering tracks and using different AI “personas” [19]. Another tool based on a transformer model is Mubert (Mubert Inc.), which allows the user to use an image instead of text as a prompt. It also offers some other parameters such as the type (track, loop, mix or jingle) and the duration of audio to be generated [20]. Beatoven (Beatoven Private Limited) is a text-to-music tool based on a generative adversarial network (GAN) model. It provides a simple interface with no other editing functionalities [21]. AIVA (Aiva Technologies SARL) is a long short-term memory (LSTM) model-based music generator, but instead of using a text-to-music approach, this tool generates chord progressions, melodies and other musical factors and allows edition of the generated output. In contrast to the above-mentioned tools, AIVA resembles a digital audio workstation (DAW) more, allowing the creator to add layers and effects, change key signatures and mix tracks [22]. Stable Audio (Stability AI Ltd) is a diffusion model text-to-music-based generator with various parameters to manipulate, namely duration, number of steps, seed and prompt strength. It also allows users to apply a previously generated result or recorded audio as an input [23]. All these music generators offer a free model with limited downloads and functionalities and do not require high musical proficiency or media expertise.

### The interplay of vision and hearing in perception

Humans have developed a highly receptive, closely integrated set of senses that serve as tools to perceive the reality surrounding them. They mostly rely on the two most well-developed ones: vision and hearing, with their relationship being indexical, which means that they support each other as hearing complements and explains the visual input [24]. Marshall and Cohen [25] formed the foundation of later media perception research by confirming that music generates meaning, establishing the role of formal congruencies between music and the visible and their associations, formed by different ways of addressing these two domains. Research suggests that the mechanisms behind congruence are inherent, as even 4-month-old infants direct greater attention to congruent stimuli [26]. On the other hand, some authors have argued that perceived congruence is a product of enculturation due to the global dominance of Western cinema and Hollywood [27]. However, the results of studies that used motion pictures as visual stimuli suggest that the majority of viewers choose the musical score intended by the composer as the most appropriate and that the choice of the musical score can manipulate the meaning of the presented plot [28–30].

Several studies provide proof of the perceptual effect of sound on vision [25,29,31–41], however the most prevalent perspective among Western scholars [42–45] is that vision is the most crucial sense and dominates over hearing, with an estimation that three-quarters of all external information provided by sense perception comes from eyesight [46].

### The present study

In the present study, we set out to investigate whether new AI-powered music creation tools can achieve the same emotional impact as human-created music in an audiovisual context. To do this, we presented the same videos with different audios, created by AI and created by humans, to three different groups of subjects. We registered biometric data (pupil dilation, blink rate and galvanic skin response) with the help of an eye tracker and skin response device, and personal affective information (emotional valence, arousal, congruence and familiarity) using the Self-Assessment Manikin [47] and a questionnaire, to analyse the differences existing between the conditions.

## Materials and methods

### Participants

A total of 91 subjects participated in this study, however 3 of them were excluded (one participant was underage, one started to feel anxious mid experiment, and another had to leave owing to an urgent business call); the remaining 88 completed the study (*N* = 88). The mean age of the participants was 29.5 years (SD 12.421 years), 41 (46.6%) were male, 47 (53.4%) were female and none identified as “other.” Just over half (*N* = 45, 51.1%) of the participants were university students, 19 (21.6%) were media professionals and the rest had other professions. Fifty-six participants (63.6%) did not use visual correction, 24 (27.3%) wore glasses and 8 (9.1%) participants wore contact lenses. Forty-five (51.1%) participants did not have any experience in playing a musical instrument, 37 (42.1%) had basic knowledge and 6 (6.8%) were very competent in playing a musical instrument. Seventy-eight (88.6%) participants were right-handed, 9 (10.2%) participants were left-handed and 1 (1.1%) participant was both-handed. Participants were randomly distributed in one of the three different conditions that we considered: (1) video with human-created music (HCM), (2) video with AI-created music based in a keyword prompt (AI-KP) and (3) video with AI-created music composed from a prompt based on a dimensional scale (AI-DP), resulting in 29 participants in the HCM condition, 29 participants in the AI-KP condition and 30 participants in the AI-DP condition. Find the distribution of participants in Table 1.

**Table 1:** Demographic data of participants, divided by conditions.

	HCM (*N *= 29)	AI-KP (*N* = 29)	AI-DP (*N* = 30)
**Gender**	**15 males, and 14 females**	**13 males, and 16 females**	**13 males, and 17 females**
Age	27.931 ± 10.687	30.345 ± 12.257	30.2 ± 14.277
Nationality	22 Spanish, 1 Egyptian, 1 Polish, 1 Chinese, 1 Venezuelan, 1 Chilean, 1 Bolivian, and 1 Turkish	23 Spanish, 1 Bolivian, 1 Peruvian, 1 Costa Rican, 1 American, 1 Brazilian, and 1 Colombian	23 Spanish, 2 Italian, 1 Panamanian, 2 Peruvian, 1 Chinese, and 1 Japanese
Music experience	1 very proficient, and 28 none to basic knowledge	1 very proficient, and 28 none to basic knowledge	4 very proficient, and 26 none to basic knowledge
Profession	16 student, 6 audiovisual (AV) technician, 3 journalist, 1 university professor, 1 marketing, 1 administrative worker, and 1 tourist administrator	15 student, 3 professor, 5 AV technician, 1 journalist, 3 administrative worker, 1 waiter, and 1 marketing	17 student, 6 AV technician, 2 communication, 1 professor, 1 journalist, 1 cook, and 2 administrative workers
Vision correction	21 none, 6 glasses, and 2 contact lenses	19 none, 8 glasses, and 2 contact lenses	16 none, 10 glasses, and 4 contact lenses
Hand dominance	26 right-handed, and 3 left-handed	25 right-handed, and 4 left-handed	27 right-handed, and 3 left-handed

Participants were randomly assigned to one of three conditions. The demographic characteristics of each group indicate a balanced distribution across conditions.

Participants were recruited between March 12^th^ and May 2^nd^ of 2024 through different channels, including mailing, hanging posters in the university and personal invitations in hallways. There was no compensation for the subjects’ participation, and each whole procedure took around 15–20 minutes.

### Ethical approval

Experiments were carried out following the guidelines, procedures and regulations for human research of the University Autònoma de Barcelona and approved by its Ethics Commission for Research with Animals and Humans (CEEAH/CERec; file number 6370), in accordance with the tenets of the Helsinki Declaration and of other relevant international codes and guidelines. Participants gave prior written informed consent to participate in the study.

### Stimuli: videos

For the video stimuli, 14 excerpts from videos were gathered from the video sharing platform Vimeo. The excerpt videos were chosen pseudo-randomly but trimmed to fit the maximum targeted length of 23 seconds. To avoid focusing on a specific genre, we selected a diverse set of videos, ensuring that variability was evenly distributed across all conditions so that any observed differences could be attributed to the musical manipulation rather than the content itself. So, the videos were of various genres and production techniques; half of them depicted facial expressions of people, whereas half of them did not. The videos were all presented in 1080p, and the original audio was removed using iMovie [48]. All 14 stimuli were randomly presented to each participant. The stimuli presented the following content: (V1) A documentary-style video depicting animals in their natural habitat [49]; (V2) a black and white abstract video depicting flowers and a broken mug [50]; (V3) a short video showing a boy dangerously handling an axe, creating suspense [51]; (V4) an AI-generated surrealistic video with gruesome motives [52]; (V5) an awareness-spreading type of video of a girl covering facial bruises with makeup [53]; (V6) a promotional video of two supercars on a racetrack [54]; (V7) an animated video of a plane and a drone flying [55]; (V8) a trailer for a dark, ominous movie [56]; (V9) a glitchy animated video, alternating between an old man and the Joker [57]; (V10) a sports edit-style video of a freeride skier [58]; (V11) a time-lapse video of nocturnal natural scenery [59]; (V12) an animated, tunnel vision-style video of space [60]; (V13) an animated, tunnel vision-style video of technological motives [61]; (V14) an advertisement for ice cream [62].

### Pre-test

To create the soundtracks, we conducted a pre-test (pilot study) with *N* = 10 participants (mean age 31 ± 18.944; 5 female; all with none to basic musical knowledge), showing the video stimuli in a silent condition to determine the emotional significance and degree of arousal for each. After each video, the participants were shown the Self-Assessment Manikin (SAM) [47] for emotional valence and arousal (with a Likert scale from 1 to 9). They were also asked which of the six basic emotions (happiness, sadness, anger, surprise, disgust and fear) was the most appropriate to describe the video. After that, they were asked to describe the video with one word (keyword) and which mood, style or musical genre would be the most appropriate as a soundtrack for the video shown. All these questions were answered verbally after each video presentation. The data from our pre-test included: mean average ratings of valence and arousal (for example, valence of 3.5 and arousal of 6.7); mean categorical emotions (in percentage); video content keywords, with a ranking number to signify the amount of times a keyword was mentioned (for example, joker (3), disturbing (1), horror (2), etc.); and appropriate soundtrack keywords with the same ranking number (for example, pop (1), romantic (2), slow (3), etc.). The results of this pre-test were used to design an appropriate soundtrack for the video as explained below.

### Stimuli: soundtracks

This experiment aimed to compare soundtracks made by humans versus those created with AI, and, as mentioned above, three different soundtrack conditions were made: one human-made and two AI-made. As explained earlier, the soundtracks were designed based on the pre-test.

#### Human-created music condition (HCM).

In the first condition, the soundtracks for the videos were chosen from a database of emotional movie soundtracks, all of which were human-made. These soundtracks were selected from the study of Eerola and Vuoskoski [63]. The soundtrack excerpts in the database are annotated using both dimensional (valence and arousal) and discrete (specific emotion) ratings. Both categories are rated on a Likert scale from 1 to 9. To find the most appropriately rated excerpts for our videos within the database of 110 songs, we used Chat GPT-4 to navigate through the data files of the said database, using the Euclidian distance for each video rating [64]. Note that since the task was to identify the entry from the database that was closest, the output would be identical in case we replicated this. On the basis of our pre-test, we used the categorical emotion (in cases where reported categorical emotion was less than 50%, we did not use it in the prompt, owing to the small sample size) and the ratings of valence and arousal. As energy and tension are correlated [63], we asked Chat GPT-4 to combine these two categories into one under the term “arousal” [64]. Using this method, we chose soundtracks for all 14 videos. Note that, despite using AI for choosing the soundtracks, the soundtracks themselves were all human-made, specifically composed for films.

#### AI music – keyword prompt condition (AI-KP).

**Fig 1:**
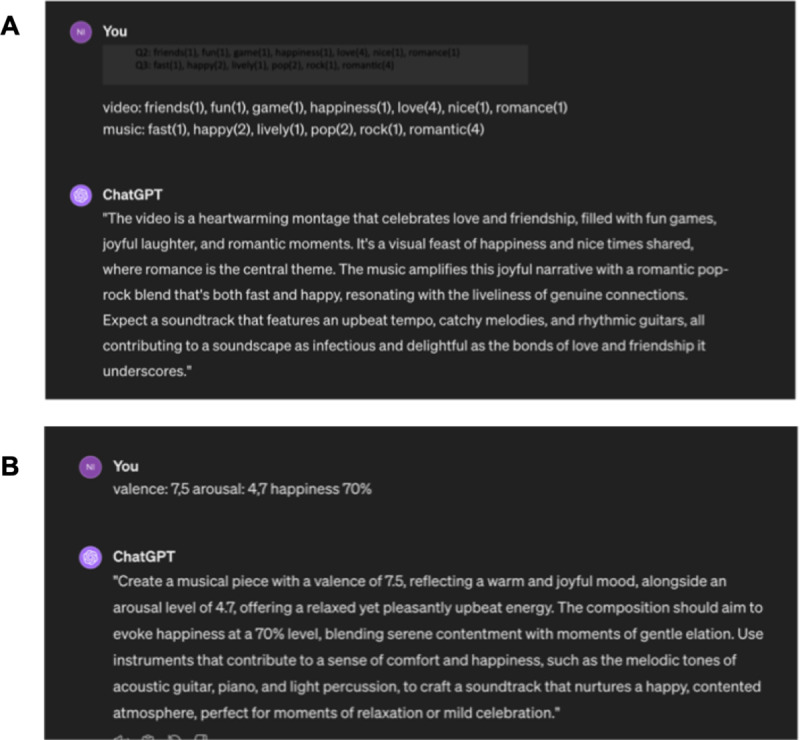
Screenshots of sample prompts used for the AI-KP and AI-DP conditions. (A) Sample prompt used for the AI-DP condition. (B) Sample prompt used for the AI-DP condition.


In the second condition, one version of AI-generated soundtracks was made using prompts based on the keywords from the pre-test. In some cases, the answers were more homogeneous, but in other cases, less so, thus we decided to rate the keywords according to their frequency of use, as mentioned above. The AI generator used was Stable Audio [23] for the following reasons: since the soundtracks from the databases did not contain lyrics, and since lyrics can convey meaning, are referential and evoke emotions by themselves [65], we avoided AI music generators that generate songs with lyrics. Moreover, with Stable Audio, the length of the generated song can be specified before being generated, so that it fits the length of the video. Other parameters available are the prompt strength (which defines how closely the generator follows the prompt), steps (where a higher step number means greater audio processing), seed (the noise arrangement used to generate the audio) and the number of results. The prompt strength was set to 50%, the step count to 50 and the seed to random, with one result generated per prompt. The prompt guide of Stable Audio provides a few tips for creating a good prompt: to add detail, such as genres, instruments and moods and to set a specific tempo [23]. Since those categories were not necessarily stated in the results of the pre-test and to minimise the amount of human agency, we asked Chat GPT-4 to generate good prompts based on our input [64]. The prompt would consist of the following form (see Fig 1A for an example):

$$\begin{array}{c} Prompt:\;(P1)video\;descriptive\;keywords\;+\;(P2)mood/style/genre\;keywords\;+\;(P3)musical\;specifications \end{array}$$

The musical specifications would be decided by Chat GPT-4 on the basis of the previous two parts. The music resulting from this protocol was used as the AI-KP condition.

#### AI music – dimensional prompt condition (AI-DP).

In the third condition, another version of AI-generated soundtracks was made, but instead of keywords, we used a simpler and less detailed approach, using the discrete and dimensional emotional values from the pre-test as an input to generate a prompt. We still used Stable Audio as in AI-KP, for the same reasons. After initially instructing Chat GPT-4 to interpret the following prompts in musical terms as in the context of a video soundtrack, we provided the ratings from the pre-test of, first, the emotional valence, then, the arousal rating and, finally, the discrete values of the most prevalent emotions with the percentage of homogeneity. Therefore, there was no contextual input in terms of the video content or musical expression. The prompt consisted of the following form (see Fig 1B for an example):


$$\begin{array}{c} Prompt:\;(P1)emotional\;valence\;rating\;+\;(P2)arousal\;rating\;+\;(P3)\%\;of\;chosen\;discrete\;emotion \end{array}$$

Since the data acquisition period (March and April 2024), the model underlying the AI music generation tool Stable Audio has been upgraded to a newer version, with results being (to our assessment) of significantly higher quality. Furthermore, other tools such as Suno [19], which we also took into consideration but disregarded as we did not want to include music with vocals, have significantly upgraded their model and included an instrumental-music-only version. To our knowledge, Stable Audio was, at the time, the most advanced tool for generating strictly instrumental music.

In all three conditions, since some of the soundtracks were shorter or longer than the videos, we edited them so that they fitted the video in terms of length – for soundtracks that were too short, we looped a part of the composition, while for those that were too long, we trimmed them and added a fade-out effect. Also, we standardised the volume of all soundtracks to ±1 dB, to avoid any potential emotional effect due to loudness. For this, we used the audio editing software foobar2000 [66]. We used the video editing software iMovie [48] to merge the soundtracks with the videos.

### Procedure

**Fig 2:**
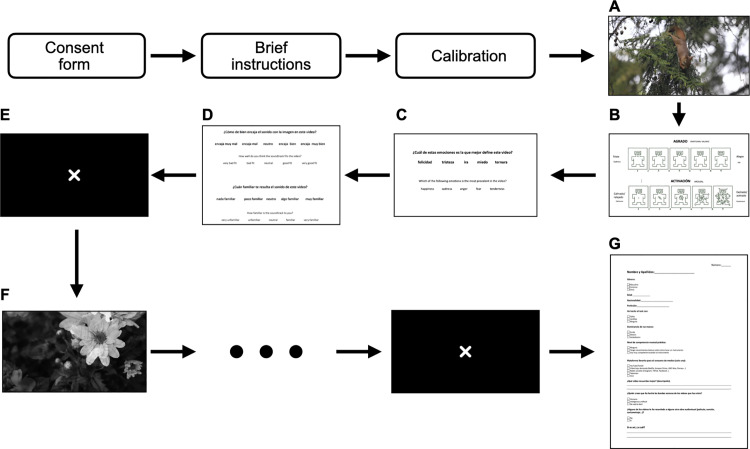
Procedure of the experiment. (A) Video stimulus; (B) Self-Assessment Manikin; (C) discrete emotions; (D) congruence and familiarity; (E) blank focus screen; (F) next video stimulus; (G) general questionnaire.

**Fig 3:**
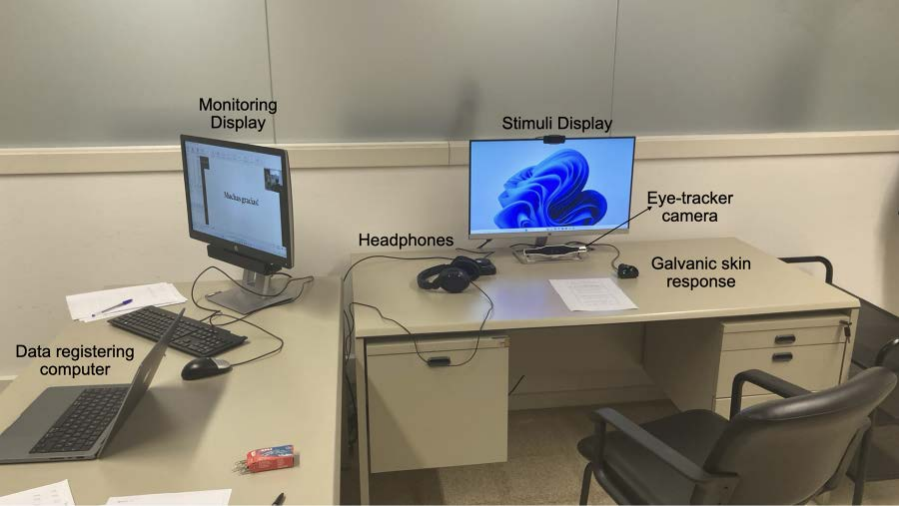
Setup of the laboratory. The setup includes a data registering computer, monitoring display, headset, stimuli display, eye-tracking camera and skin response and heart rate device.

The experimental part of our study was carried out mostly at the Neuro-Com Laboratory at Universitat Autònoma de Barcelona and partly at the Spanish Public Television Institute (RTVE), in Barcelona, Spain. As mentioned above, the participants were distributed randomly to one of the three conditions: HCM, AI-KP and AI-DP. Once each participant entered the laboratory, they signed the consent form and were asked to sit in front of the screen. After that, participants received brief instructions for the experimental session, which included explanations of Figs 2B, 2C, and 2D related to the questions they would answer after each stimulus presentation. Then, we put their hand into the galvanic skin response (GSR) device and calibrated their gaze with the eye-tracker device (see the ‘Technical specifications’ section for more detail). Once their gaze had been calibrated, the videos were shown in a randomised order to avoid any bias due to stimuli fatigue. After each video, questions regarding the emotional valence, arousal, discrete emotion of the video, congruence of the soundtrack and familiarity of the soundtrack were shown on the screen. The participants responded to the questions verbally to avoid losing their gaze calibration during the experimental part. After answering the questions, a cross was shown for 5 seconds in the middle of the screen. The procedure for each participant lasted approximately 15 minutes. After all the videos had been shown along with their respective questionnaires, the participants were asked to answer some general questions regarding their age, profession, nationality, dominant hand, use of vision correction devices, level of practical musical proficiency, favourite platform for media consumption, most memorable video among the 14, how they think the soundtracks were created (human or AI) and if any of the stimuli shown reminded them of any previously seen media (and, if yes, which previously seen media). The whole procedure is illustrated in Fig 2. Our setup included a table and chair for the participant, positioned in front of the screen, and a separate table and chair for the researcher, allowing them to monitor the stimulus display and ensure the experiment was proceeding correctly. The lab was settled in a basement with no visual and auditory disturbances. A picture of our setup is shown in Fig 3.

### Technical specifications

For registering the data, we used a HP ProDesk 600 G5 MT computer with an Intel Core i7 processor (3.20 GHz, 16 GB RAM), which was above the technical requirements of the eye-tracking camera (2.40 GHz). For the eye-tracking and biometrical data, we used a Gazepoint 3 (Gazepoint) biometrics kit, which includes an eye-tracking camera and a separate compartment for measuring the galvanic skin response and heart rate. The software used for the eye-tracking and biometrics hardware was Gazepoint Analysis (with Gazepoint Control for calibration). The refresh rate for the biometrics was set to 60 Hz. Overall, the Gazepoint 3 biometrics kit is assessed as reliable and valid for psychophysiological research [67]. The monitor used for displaying the stimuli was 27 inches (HP 27fw). To ensure that the audio quality was as good as possible, we used Sennheiser HD-200 Pro studio headphones with an impedance of 32 ohms, frequency range of 20–20,000 Hz and sound pressure level of 108 dB. The volume of the stimuli on the computer was set to 30% in all cases. The distance between the display and the participants’ eyes was approximately 60 cm in all cases.

### Data acquisition and analysis

The biometrical data were acquired using the eye-tracker and biometrics kit, and the self-reported data were acquired using a questionnaire. For the biometrical data, three different variables were acquired: (1) pupil dilation, as an indicator of emotional state during exposure to the audiovisual stimuli [37], with a higher pupil diameter signifying a higher state of arousal [68]; (2) blink rate, as an indicator of aversion towards the presented stimuli, with a higher blink rate generally indicating a higher level of aversion [69], while conversely, it can also indicate a lower level of cognitive workload and therefore a higher state of relaxation while exposed to stimuli [69,70]; and (3) galvanic skin response, which can indicate the state of arousal, with a higher skin conductance level resulting in a lower resistance level, which can be interpreted as heightened arousal [71]. For the self-reported data, five elements were acquired: (1) emotional valence, measured using a Self-Assessment Manikin on a scale from 1 to 9; (2) arousal, measured using a Self-Assessment Manikin on a scale from 1 to 9; (3) the most prevalent discrete emotion among the five most often associated in music (happiness, sadness, tenderness, anger and fear), which we chose on the basis of previous studies carried out by researchers [63,72,73]; (4) perceived congruence or audiovisual fit, measured on a Likert scale from 1 to 5), with a higher congruence linked with a heightened emotional valence [25]; and (5) perceived familiarity of the soundtrack, measured on a Likert scale from 1 to 5, positively correlated with higher emotional valence [74].

To assess whether the data were normally distributed, we conducted a Shapiro–Wilk test, which revealed that, except for the most prevalent discrete emotion, none of the data were normally distributed. Therefore, we used a Kruskal–Wallis *H* test instead of an analysis of variance (ANOVA) to determine whether there were statistically significant differences depending on the audiovisual condition. We carried out post hoc analysis using Dunn’s test.

## Results

### Biometric data

#### Pupil dilation.

**Fig 4:**
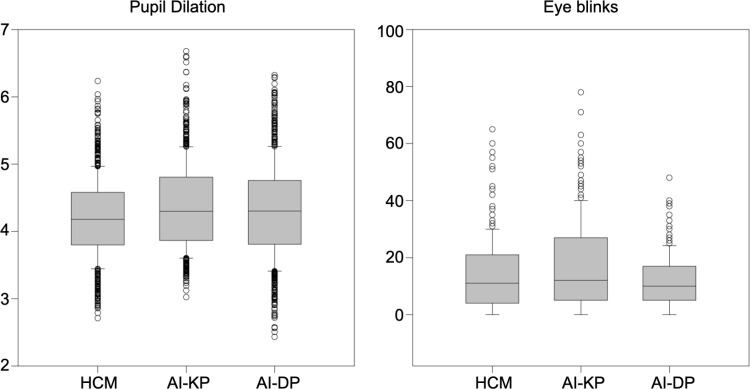
Boxplot of pupil dilation and eye blinks. The data points include the subgroups for the three studied conditions: HCM (human-created music condition), AI-KP (AI music – keyword prompt condition) and AI-DP (AI music – dimensional prompt condition).

For pupil dilation of both pupils together, we used the eye-tracking camera, continuously measuring the pupil diameter while presenting the stimuli. The results (Fig 4) were the following (in mm): HCM = 4.196 ± 5.953, AI-KP = 4.374 ± 6.478 and AI-DP = 4.312 ± 7.108; the mean ranks of pupil dilation were statistically significant among conditions, *H*^2^(2) = 22.072, *p* < .001. Post hoc analysis using Dunn’s test revealed significant differences between HCM and AI-KP (*z* = 4.530, *p* < .001) and between HCM and AI-DP (*z* = 3.320, *p* < .001). There were no significant differences between AI-KP and AI-DP (*z* = 1.231, *p* = .218). The results indicate that both AI-generated soundtrack conditions resulted in wider pupil dilation than HCM, but they did not differ significantly between each other.

#### Blink rate.

For blink rate, we used the eye-tracking camera, which gave us the number of blinks per stimuli, and to get a standardised blink rate, we calculated the rate per minute, according to the duration of a specific stimulus. The results (Fig 4) were the following (in blinks per minute): HCM = 14.052 ± 12.245, AI-KP = 16.826 ± 15.617 and AI-DP = 11.645 ± 19.031; the mean ranks of blink rate were statistically significant among conditions, *H*^2^(2) = 10.077, *p* = .006. Post hoc analysis using Dunn’s test revealed significant differences between AI-KP and AI-DP (*z* = 3.163, *p* = .002). This indicates that the AI conditions differed between each other, with AI-KP generating a significantly higher blink rate, but not compared with HCM.

#### Galvanic skin response.

**Fig 5:**
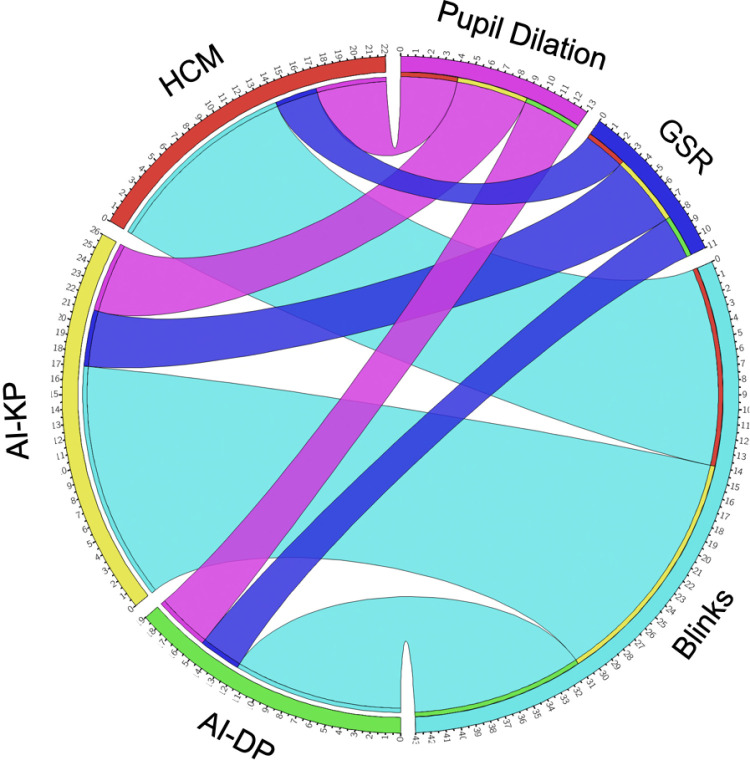
Chord diagram depicting the interactions among the recorded biometric data. The data include pupil dilation in pink (in mm), blink rate in blue (blinks per minute) and GSR in purple (in mΩ) in the three studied conditions: HCM (human-created music condition) in red, AI-KP (AI music – keyword prompt condition) in yellow and AI-DP (AI music – dimensional prompt condition) in green. Notably, among the three biometric indicators analysed (pupil dilation, GSR, and blinks), the AI-KP condition consistently produced the highest response levels.

For galvanic skin response (GSR), we used the device that measured the skin impedance level in ohms, which was measured consistently during the whole duration of our experiment. The results (Fig 5) were the following (in ohms): HCM = 344,598.811 ± 448,607.922, AI-KP = 438,757.594 ± 380,518.129, and AI-DP = 287,474.815 ± 274,982.562; the mean ranks of GSR were statistically significant among conditions, *H*^2^(2) = 71.381, *p* < .001. Post hoc analysis using Dunn’s test revealed significant differences between AI-KP and AI-DP (*z* = 7.929, *p* < .001) and between AI-KP and HCM (*z* = 6.488, *p* < .001). This indicates that AI-KP resulted in a significantly higher skin impedance level, being less arousing than the other two conditions.

### Self-reported data

#### Emotional valence.

To establish whether there was a significant difference between the three presented conditions, we used the Self-Assessment Manikin (SAM), which serves as a tool for conscious measurement of emotional valence on a scale from 1 to 9. This metric was shown on the screen after each presented stimulus. The results were the following: HCM = 5.443 ± 2.396, AI-KP = 5.387 ± 2.43 and AI-DP = 5.479 ± 2.191; the mean ranks of valence were not statistically significant among the three conditions: *H*^2^(2) = 0.093, *p* = .955.

This implies that there are no differences among the conscious sentiment towards the presented stimuli depending on the soundtrack.

#### Arousal.

For self-reported arousal, we also used the SAM scale, with a scale of arousal from 1 to 9. As for the previous metric, this one was shown on the screen after each presented stimulus. The results were the following: HCM = 4.933, ± 2.270, AI-KP = 5.397 ± 2.163 and AI-DP = 5.555 ± 2.035; the mean ranks of arousal were statistically significant among conditions: *H*^2^ (2) = 16.039, *p* < .001. Post hoc analysis using Dunn’s test revealed significant differences between HCM and AI-KP (*z* = 2.906, *p* = .004) and between HCM and AI-DP (*z* = 3.845, *p* < .001). The conditions AI-KP and AI-DP did not differ significantly (*z* = −0.915, *p* = .360).

This implies that the participants perceived the soundtracks in the conditions AI-KP and AI-DP as significantly more arousing than their human-made counterpart, HCM.

#### Most prevalent discrete emotion.

For self-reported perceived emotion of the videos, we used a previously mentioned set of five different musically felt emotions (happiness, sadness, tenderness, anger and fear), which were presented to the participants after each stimulus. We calculated the percentage homogeneity in choosing the same emotion across the three studied conditions. The results were the following (in %): HCM = 70.193 ± 15.815, AI-KP = 73.893 ± 12.895 and AI-DP = 70.714 ± 15.521; the mean ranks of perceived emotion were not statistically significant among conditions: *F*(2, 39) = 0.256, *p* = 0.775. This means that the most perceived emotion in the videos did not differ depending on the supporting soundtrack condition.

#### Perceived congruence.

For the perceived congruence of the video and sound in the stimuli, we used a Likert scale from 1 to 5, which was presented to the participants after each stimulus. The results were the following: HCM = 3.564 ± 1.070, AI-KP = 3.761 ± 3.761 and AI-DP = 3.433 ± 1.109; the mean ranks of perceived congruence were statistically significant among conditions: *H*^2^(2) = 19.110, *p* < .001. Post hoc analysis using Dunn’s test revealed significant differences between AI-KP and AI-DP (*z* = 4.334, *p* < .001) and between AI-KP and HCM (*z* = 2.678, *p* = .007). This means that the AI-KP condition scored significantly higher in terms of congruence than AI-DP and HCM.

#### Perceived familiarity.

**Fig 6:**
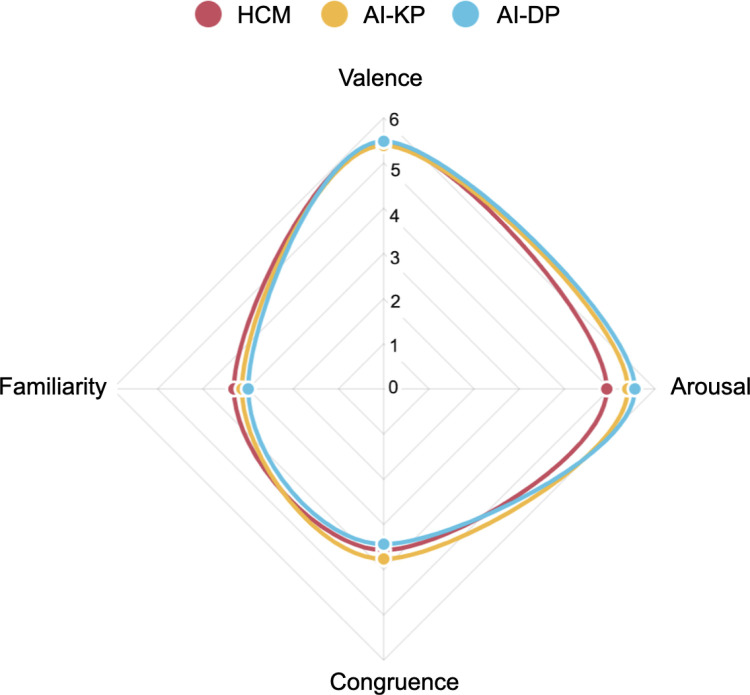
Radar spider chart of self-reported data. The data include the valence, the arousal, the congruence and the familiarity in the three studied conditions: HCM (human-created music condition), AI-KP (AI music – keyword prompt condition) and AI-DP (AI music – dimensional prompt condition).

For the perceived familiarity of the soundtrack in the stimuli, we used a Likert scale from 1 to 5, which was presented to the participants after each stimulus. The results were the following: HCM = 3.311 ± 1.033, AI-KP = 3.133 ± 1.071 and AI-DP = 2.995 ± 1.034; the mean ranks of perceived familiarity were statistically significant among conditions: *H*^2^(2) = 19.101, *p* < .001. Post hoc analysis using Dunn’s test revealed significant differences between HCM and AI-KP (*z* = −2.623, *p* = .009) and between HCM and AI-DP (*z* = −4.342, *p* < .001), which means that the soundtrack behind the HCM condition was perceived as significantly more familiar than both AI conditions (Fig 6).

## Discussion and conclusions

This work aimed to explore the emotional impact of AI-generated music versus human-composed music in audiovisual experiences through biometric and self-reported analyses. To do so, we created three groups of study that watched the same videos but with music of different origins: (1) created by humans, (2) created by an AI system using more detailed and sophisticated prompts based on keywords and (3) created by an AI system too, but using simpler and less detailed prompts based on discrete and dimensional emotional values.

We found that both types of AI-generated music led to wider pupil dilation and were identified as more arousing compared with the human-created music. The pupillary response reflects attentional modulation to sound after emotional arousal [75]. Since pupils dilate as a consequence of attentional effort [76], our findings suggest that decoding the information from AI-generated music may require greater effort. Considering that the AI-based soundtracks were also scored with lower familiarity than human-made music, we could suggest that, overall, in audiovisual contexts, AI-generated music tracks would require more resources to decode than human-created music.

So, as could be expected, human-created music was revealed as more familiar to participants. Familiar visual objects are processed faster and more accurately than unfamiliar objects [77], and familiarity is basic in marketing decision-making [78,79]. We did not ask participants to rate their preference regarding the type of soundtrack, since each individual only listened to one out of the three conditions, but on the basis of previous studies that have found a significant relationship between familiarity and preference in music [80], our result may indicate a stronger preference for human-created music. In Western musical composition—particularly in music intended to support visual content—there are established conventions that are frequently repeated. Even in mainstream music, similar patterns emerge in the use of sonic properties such as tonality, instrumentation, rhythm, and tempo. In contrast, AI-generated content (including images, music, text, and video) is often reported to evoke an “uncanny” aesthetic, producing an eerie or even uncomfortable feeling [81]. While some argue that AI-generated media is merely a “remix” of existing material, in the context of background music, novelty and uniqueness are secondary to the primary goal of effectively supporting and enhancing the visual experience. Therefore, we suggest that the perceived familiarity of the human-composed condition may stem from the established musical conventions commonly used in Western cinematic composition. On the other hand, the finding that human-created music was perceived as more familiar—and was associated with lower arousal and reduced pupil dilation—could suggest that processing familiar content is less cognitively demanding than processing novel material.

The origin of the music (AI versus human) was not relevant in terms of attention as analysed by the viewers’ blink rate. Eyeblinks are correlated with cognitive load during visual and auditory paradigms [82–85]. Previous works have shown that the content of audiovisuals [86,87] and the style of edition in audiovisuals [88,89] do affect viewers’ blink rate, suggesting the impact that those two variables (content and style) have on viewers’ attention. Here, however, we did not find that the origin of the music was relevant when comparing AI versus human origin. On this basis, we suggest that the same attention can be obtained regardless the origin of the music and that using AI-generated music does not affect viewers’ cognitive load compared with using human-made music.

The percentage of the most prevalent discrete emotion found in each video was similar regardless of the type of soundtrack, and the emotional valence in the three conditions did not show significant differences either. So, on the basis of the two strategies used to study emotions in this experiment, one can conclude that the origin of the soundtrack, whether AI or human-made, does not affect the emotion elicited in the viewers. Several previous works have studied the role that auditory elements may play in emotional perception [90], but on the basis of our results, we suggest that the type of creator behind a soundtrack (AI system or human) is not relevant in this regard. We conclude that the lack of greater differences between human-made and AI-created music when watching audiovisual works could be related to the greater domination that vision has over hearing [42–45].

The galvanic skin response showed that one of the AI conditions (AI-KP) resulted in a higher skin impedance level. The fact that the condition with the lowest skin impedance level was the other AI condition (AI-DP) indicates that, apparently, the origin of the soundtrack (AI or human) is not a relevant variable, either. Interestingly, the perceived congruence and blink rate showed similar results, with higher congruence and blink rate found in the AI-KP condition and the lowest congruence and blink rate in the AI-DP condition. Potential reasons for this could be stress and cognitive workload needed for decoding less congruent stimuli. As higher skin response is correlated with lower arousal [71], higher blink rate is correlated with lower attention [69,86] and a higher perceived congruence is correlated with a relaxing response [25] (due to less cognitive strain necessary for processing the two simultaneous mediums), our results suggest that these three metrics can be studied together to interpret viewers’ engagement and workload towards audiovisual content. Further studies could approach this correlation between blinks, GSR and perceived congruence found in our work. Moreover, the fact that higher skin response found in AI-KP does not correlate with lower self-reported arousal could be due to several reasons such as social desirability bias, social desirability bias, lack of emotional self-awareness, laboratory anxiety, among others [91,92].

Finally, note that interpreting physiological data would not always completely reflect emotional states or the reasons for them. Moreover, as seen, some biometrics have been correlated with different emotional states, therefore, we should acknowledge that our results may be ambiguous.

## Limitations and future studies

While this study offers valuable insights into emotional impact of AI-generate vs. human-composed music in audiovisuals, several limitations should be acknowledged to contextualize the findings and guide future research. We did not ask participants about their clinical conditions (especially neurological conditions) that could have affect their answers. Another limitation of this study is the small number of participants in the pre-test (N = 10). Future pre-tests should include larger and more diverse samples to improve the generalizability and robustness of the findings. Another limitation of this study is that some of the videos [51,60] used as stimuli are no longer accessible online. This affects the replicability and transparency of the research, as future researchers cannot review the original materials.

Moreover, we acknowledge that it is difficult to generalize the findings of our study in regard to the properties of AI. For the experiment, we used only one AI generator (Stable Audio), however, to assess of the general capabilities of large language model (LLM) based audio generators, we would have had to use other models such as Suno, AIVA, Udio, or Beatoven. Moreover, all of the mentioned generators have progressed greatly since this study was carried out. Therefore, one of our limitations is the non-generalizability of our findings as to what AI audio generators are capable of.

In addition, even our rationale for using a between-subjects design was to avoid potential carryover effects, familiarity bias, and emotional fatigue, the fact that each participant was exposed only to one of the conditions limited our ability to draw conclusions about individual subjective preferences or direct emotional comparisons across conditions. Also, although we collected participants’ perceived familiarity with the music, we did not assess prior exposure or recognition of the specific excerpts, which may have influenced emotional responses and represents a potential confounding factor.

Furthermore, while psychophysiological metrics can be good indicators of emotional impact, the observed changes are not always interpretable universally. Cognitive effects on media perception are highly complex processes, which are based on a combination of physiological, cultural and situational factors. Changes, observed with the eye tracker are especially ambiguous, since the pupil and eye behavior in general can indicate different emotional states. For a more thorough understanding of the perception of our stimuli, we should also include other methodological approaches such as EEG (which helps understand in more detail the cognitive processing and the reaction to audiovisual media) and interviews (which helps understand the cultural background, media consumption habits, personal preferences and more meaningful assessment of the sentiment towards the shown stimuli).

In future studies, we could include comparisons between laypersons and musicians to explore potential differences related to musical training and professionalization. Moreover, we could examine whether the matching of AI-generated music remains consistent across different experimental samples. Additionally, in future research, rather than selecting human-composed music from existing databases, we plan to collaborate with professional composers to create original pieces. This will allow for a more direct comparison between human-composed and AI-generated soundtracks—a direction we are already actively exploring.
